# Optimal cutoff value for assessing changes in intrahepatic fat amount by using the controlled attenuation parameter in a longitudinal setting

**DOI:** 10.1097/MD.0000000000013636

**Published:** 2018-12-14

**Authors:** Sang Bong Ahn, Dae Won Jun, Bo-kyeong Kang, Mimi Kim, Misoo Chang, Eunwoo Nam

**Affiliations:** a Department of Internal Medicine, Nowon Eulji Medical Center, Eulji University School of Medicine; b Department of Internal Medicine, Hanyang University School of Medicine; c Departments of Radiology; d Biostatistical Consulting and Research Laboratory, Hanyang University, School of Medicine, Seoul, Korea.

**Keywords:** controlled attenuation parameter, fatty liver, hepatic steatosis

## Abstract

The controlled attenuation parameter (CAP) has shown a good correlation with the intrahepatic fat amount in cross-sectional studies. However, there is no study on whether the change of CAP scores can also show good correlation in a longitudinal setting. Therefore, we investigated the correlation between CAP and magnetic resonance imaging-estimated proton density fat fraction (MR PDFF) through serial examination in a longitudinal setting.

Sixty-five patients with nonalcoholic fatty liver disease were evaluated with MR PDFF and transient elastography including CAP at baseline and 3 months later.

The CAP and MR PDFF at baseline showed a strong correlation in assessing hepatic steatosis (r = 0.66, *P* < .001). After treatment, the correlation between the change in CAP after treatment and the intrahepatic fat change (%) on MR PDFF was not satisfactory (r = 0.37, *P* = .005) in the longitudinal setting. The optimal cutoff value of the change in CAP for discriminating an improvement or an aggravation in intrahepatic fat percentage (>1% change in MR PDFF) was selected as 38 dB/m (area under the receiver operating characteristic curve = 0.559). For CAP changes > 38 dB/m, the predictive value was 14/16 (87.5%), whereas for changes < 38 dB/m, the predictive value was 12/41 (29.3%). Thereby, the accuracy of the method using the change in CAP was only 26/57 (46%). In addition, Cohen's kappa value was not significant (κ=0.11, *P* = .186).

Careful interpretation of the steatosis change based on the CAP score is needed when the absolute change value is < 38 dB/m in a longitudinal setting.

## 1 Introduction

Estimating the degree of steatosis and monitoring the amount of intrahepatic change are important in patients with nonalcoholic steatohepatitis (NASH), both in clinical trials and in real practice. Several studies reported that the controlled attenuation parameter (CAP) showed good diagnostic accuracy in detecting hepatic steatosis in various liver diseases.^[1,2,3]^ The CAP is well correlated with the steatosis grade in liver biopsy samples.^[4]^ Furthermore, CAP has a comparable diagnostic value for hepatic steatosis quantification to that of magnetic resonance imaging (MRI).^[5]^

Recently, MRI-based proton density fat fraction (MR PDFF) showed a good correlation with histologically determined steatosis grade in patients with fatty liver.^[6,7]^ In addition, MR PDFF showed fat information for the entire liver, whereas liver biopsy can evaluate only a small portion of the entire liver. Moreover, MR PDFF was a more sensitive quantification tool than the histological steatosis grade in the clinical trial setting.^[8]^ The MR PDFF is believed to be a reasonable method for quantifying changes in liver fat in clinical trials.

Although CAP has shown good performance in the quantitative assessment of liver fat in many cross-sectional studies, it has a large variation in some patients, especially those with severe fatty liver and obesity. Moreover, the use of CAP in measuring changes in the hepatic fat amount has not been evaluated in the longitudinal setting. Especially, when the change of hepatic fat is small, it is difficult to accurately measure the degree of change. Some clinical studies have used MR PDFF to measure the hepatic steatosis change in NASH.^[9]^ However, there are no data on whether CAP is a reliable test for the follow-up assessment of hepatic steatosis. In this study, we investigated the correlation between CAP and MR PDFF by using serial examination in a clinical trial setting.

## 2 Patients and methods

### 2.1 Study design and subjects

We conducted a randomized, double-blinded, placebo-controlled trial with the purpose of evaluating the changes in intrahepatic fat (IHF) measured by using MR PDFF. Probiotics or placebo was administered to adults with obesity for 12 weeks. The patients underwent MR PDFF evaluation before and after taking the assigned treatment. This study is a subgroup analysis to determine the role of CAP in assessing the hepatic fat change. This study was approved by the institutional review board of our institution. The study protocol followed the guidelines of the 1975 Declaration of Helsinki. The protocol was registered at the Clinical Research Information Service (http://cris.nih.go.kr/cris/index.jsp) with registration number KCT0001588.

### 2.2 Inclusion criteria

Participants ages between 19 and 75 years, with body mass index (BMI) of ≥25 kg/m^2^, were enrolled. Fatty liver was defined as a mean hepatic fat fraction (FF) of ≥5.0% measured from MR PDFF. All patients signed an informed consent form.

### 2.3 Exclusion criteria

The exclusion criteria included the presence of liver disease; comorbidity such as hepatitis B, hepatitis C, and eating disorder (anorexia, bulimia); and use of diuretics or drugs that could affect absorption, metabolism, and excretion, such as amphetamine, cyproheptadine, phenothiazine, appetite suppressants, and appetizers; and use of probiotics within 2 weeks before the screening.

Patients were also excluded if they drank ≥210 and ≥140 g/week of alcohol on average in the last 2 years, if their weight decreased by ≥10% within 6 months, and if their diet or exercise habits could influence the results of the clinical trial within 3 months after screening.

### 2.4 Biochemical and clinical parameters

Characteristics including sex; age; BMI; serum levels of aspartate aminotransferase (AST), alanine aminotransferase (ALT), gamma-glutamyl transpeptidase, total cholesterol, triglyceride, low-density lipoprotein, high-density lipoprotein, albumin, bilirubin, and fasting plasma glucose; platelet count; and prothrombin time were documented. Both MR PDFF and CAP were evaluated after 3 months of the probiotic clinical trial in patients with nonalcoholic fatty liver disease (NAFLD).

### 2.5 CAP measurement

Hepatic steatosis was measured according to CAP scores by using the M probe of the transient elastography device (Echosens, Paris, France). The CAP was evaluated by a trained investigator. Ten valid measurements were taken according to the manufacturer's recommendation. A success rate of > 60% was required for a valid measurement. An interquartile range > 30% of the median liver stiffness value was considered unreliable, and such measurements were excluded from data analysis.^[10]^ Eight of the 65 patients were excluded owing to unreliable measurements.

### 2.6 MRI examination

All of the included patients underwent 3-T MRI scans by using an MRI system with torso coil (Ingenia; Philips Healthcare, Best, Netherlands). We obtained axial T2-weighted turbo spin echo sequences (time of repetition [TR] shortest automatic, time of echo [TE] 80 ms, flip angle 90°, matrix 280 × 190, field of view [FOV] 330 × 300 mm, slice thickness 6 mm, gap 1 mm, number of slices 25, number of signal average 1, scan time 13 s). The PDFF was measured by using an mDIXON-Quant sequence (TR shortest automatic, 6 TEs [1st TE shortest automatic, delta TE 0.8 to 1.01], flip angle 3°, parallel imaging SENSE factor 2, number of signal average 1, matrix size 300 × 300, FOV 350 × 350 mm, number of slices 60, slice thickness 3 mm, scan time 14 s).

### 2.7 Hepatic fat quantification with MR PDFF

The MR PDFF was analyzed by 1 radiologist who was blinded to the clinical information of all patients and to the MRI scanning sequences. The 3 nonoverlapping circular regions of interest (ROIs) of 100-mm^2^ size were located in Couinaud's liver segment by avoiding large vessels, ducts, and image artifacts to obtain a total of 24 ROIs. Each ROI was placed such that they visually match, as much as possible according to the location of adjacent vessels in 2 MRI scans (at the baseline and after treatment). The mean of 3 values obtained from each liver segment was calculated and used as a representative value. For hepatic fat quantification, we performed MR PDFF evaluation as the reference standard. We classified the patients into S0 to S3 based on MR PDFF results (S0: least steatosis, S3: most steatosis).

### 2.8 Optimal cutoff value of CAP change for discriminating actual changes of hepatic fat

A previous study^[8]^ suggested that a > 1% change in hepatic steatosis would be reflected as an actual change in MRI examination. The diagnostic ability of CAP change for discriminating actual changes in hepatic steatosis (> 1% change in MR PDFF) was assessed by calculating the area under the receiver operating characteristic curve (AUROC). After comparing the performance of various thresholds, the optimal cutoff value of CAP change was selected for discriminating actual changes in hepatic fat.

### 2.9 Diet diary and exercise education

Subjects were instructed to record the average number of weekly or daily intake and the amount of intake per serving by food group. Afterwards, the subjects received a dietary evaluation by consulting with the investigator based on the diet diary recorded at the screening points and they were given an education on the appropriate amount of daily nutrition intake and exercise. Compliance to diet and exercise were evaluated on a 5-point Likert scale. The higher scores were the higher the compliance to diet and exercise are (1 = Completely Fail, 2 = Fail, 3 = Satisfactory, 4 = Good, 5 = Excellent). Questions on diet diary and exercise were asked using the validated questionnaire which was used in KNHANES. Patients were significantly reduced in calorie intake after diet education.

### 2.10 Statistical analysis

The baseline characteristics of patients are presented as mean ± standard deviation or median (Q1, Q3) for continuous variables, and as frequency (%) for categorical variables. The general statistics of the patients were compared between groups by using the Mann–Whitney test, Kruskal–Wallis test, or Jonckheere trend test, as appropriate. The relationship between 2 continuous variables was explored by calculating Spearman's correlation coefficients or by fitting a smoothing spline (penalized B-spline) to the data set. The predictive performance of CAP change for discrimination was assessed by calculating predictive values, accuracy, and Cohen's kappa. Statistical analyses were conducted with SAS software version 9.4 (SAS Institute Inc., Cary, NC) and MedCalc version 17.2 (MedCalc Software, Ostend, Belgium). A 2-tailed *P*-value of < .05 was considered statistically significant.

## 3 Results

### 3.1 Clinical characteristics of patients

Sixty-five patients with NAFLD were enrolled from Sep 2015 to Feb 2016. All subjects simultaneously underwent MR PDFF and CAP evaluations at baseline and 3 months later. Eight patients were excluded owing to unreliable CAP measurements. Therefore, 57 patients with NAFLD were included in the final analysis. Patients with NAFLD were classified according to the degree of steatosis as measured with MR PDFF. The clinical characteristics of patients are presented in Table 1. The sex distribution did not differ significantly among the 4 groups, and only a slight difference in weight was observed. In the laboratory findings, the lipid profile did not differ among groups; however, glucose, AST, ALT, and insulin showed an increasing trend with the increase in the degree of steatosis (Table 2).

**Table 1:**
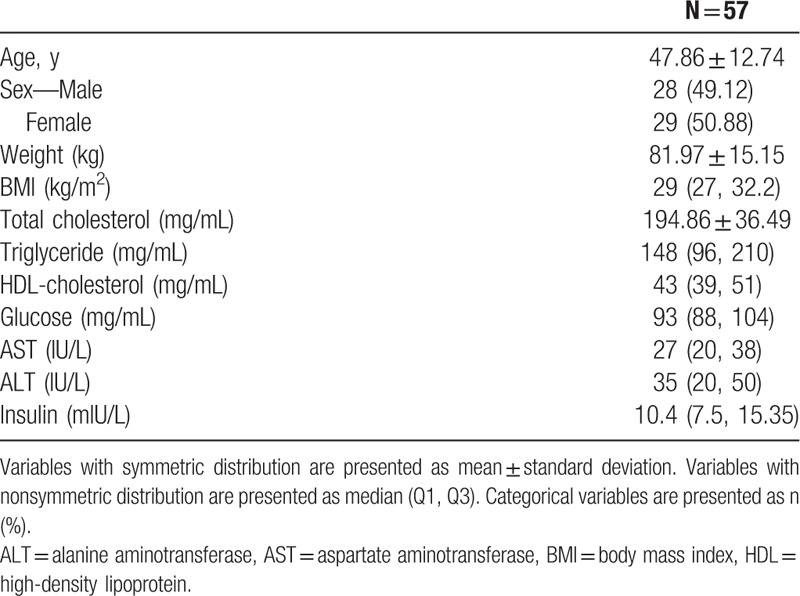
Baseline characteristics of patients.

**Table 2:**
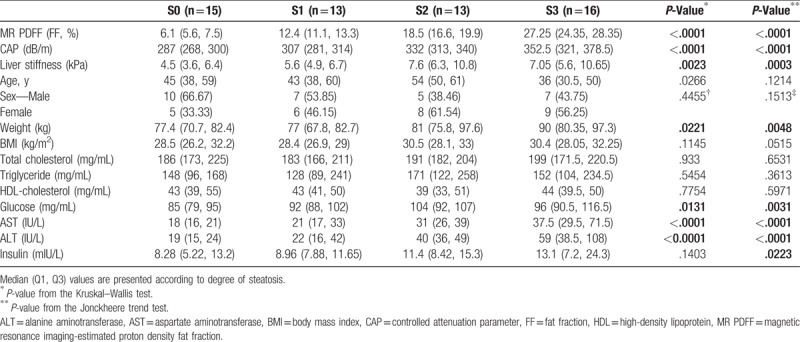
Comparison of elastography, MR PDFF, and CAP according to degree of steatosis.

### 3.2 Correlation between baseline CAP and baseline MR PDFF in the cross-sectional setting

**Figure 1:**
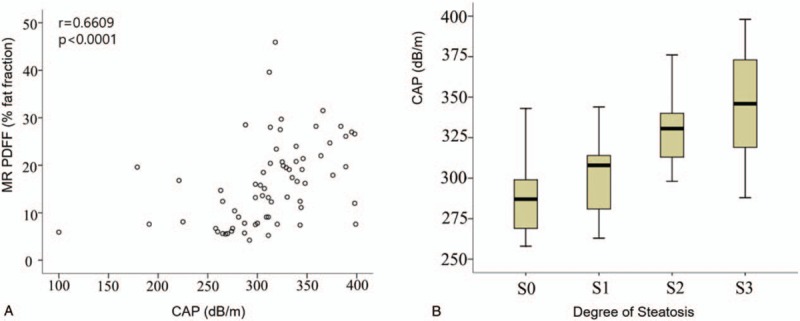
Correlation between baseline controlled attenuation parameter (CAP) and baseline magnetic resonance imaging-estimated proton density fat fraction (MR PDFF) in the cross-sectional setting. (**A**) Relationship between CAP and MR PDFF: CAP and MR PDFF achieved a moderate or strong correlation (r = 0.66, *P* < .001). (**B**) Comparison of CAP according to degree of hepatic steatosis: differences and an increasing trend of CAP values were observed according to the degree of hepatic steatosis.

The baseline CAP and MR PDFF showed a strong correlation in assessing hepatic steatosis (r = 0.66, *P* < .001) (Fig. 1**A**). The CAP and liver stiffness were significantly different among the hepatic steatosis groups (*P* < .001 and *P* < .002, respectively) and showed an increasing trend according to the degree of hepatic steatosis (*P* < .001 for both) (Table 2, Fig. 1**B**).

### 3.3 Correlation between CAP change and MR PDFF change in the longitudinal setting

**Figure 2:**
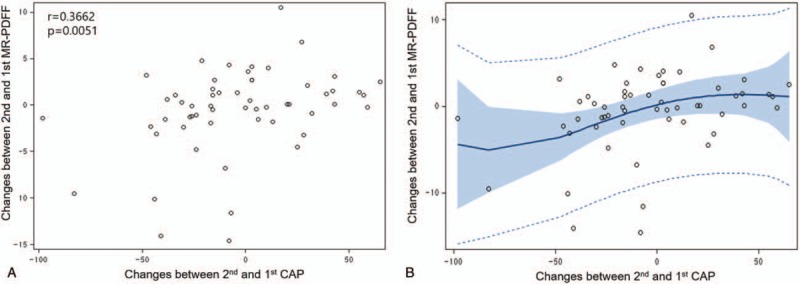
Correlation between controlled attenuation parameter (CAP) change and magnetic resonance imaging-estimated proton density fat fraction (MR PDFF) change in the longitudinal setting. (**A**) Relationship between CAP change and MR PDFF change: CAP change and MR PDFF change achieved a weak correlation (r = 0.37, *P* = .005). (**B**) Penalized B-spline: the penalized B-spline suggests that the relationship between the 2 variables is symmetric with respect to the origin (0, 0).

A significant but weak correlation was observed between CAP change after treatment and intrahepatic fat change (%) by using MR PDFF in the longitudinal setting (r = 0.37, *P* = .005). The MR PDFF change after treatment showed moderate correlations with the BMI change (r = 0.42, *P* = .001), AST change (r = 0.50, *P* < .001), and ALT change (r = 0.49, *P* < .001), and weak correlations with glucose change (r = 0.28, *P* = .032) and total body fat change (r = 0.28, *P* = .036) (Table 3). In contrast, the CAP change after treatment showed weak correlations with the BMI change (r = 0.39, *P* = .003) and total body fat mas change (r = 0.26, *P* = .049) (Table 3). In addition, the smoothing of data by using a penalized B-spline (Fig. 2**B**) suggested that the relationship between CAP change and MR PDFF change is symmetric with respect to the origin (0, 0). Thus, we focused on the changes in absolute values for both CAP and MR PDFF.

**Table 3:**
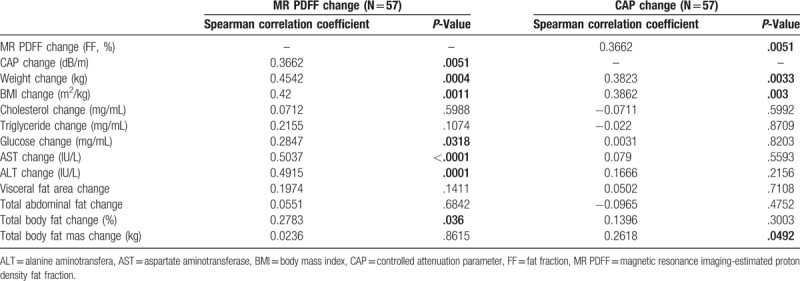
Correlation between changes in clinical parameters and MR PDFF/CAP after 12 weeks.

### 3.4 Predictive performance of CAP change for discriminating actual changes in hepatic fat

**Figure 3:**
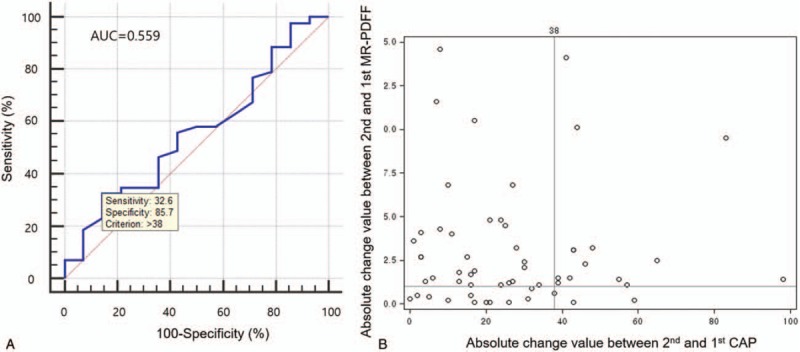
Optimal cutoff value of controlled attenuation parameter (CAP) change for discrimination. (**A**) Receiver operating characteristic curve for discriminating actual changes of hepatic fat: the optimal cutoff value of CAP change for discriminating an actual change (≥1%) of hepatic fat was selected as 38 dB/m (area under the receiver operating characteristic curve = 0.559, sensitivity 32.6%, specificity 85.7%). (**B**) Distribution of hepatic steatosis change in absolute value: the optimal cutoff values of MR PDFF change (1%) and CAP change (38 dB/m) were added to the distribution of hepatic steatosis change in absolute values.

To discriminate between an improvement or an aggravation in intrahepatic fat percentage after treatment, based on the suggested definition of an actual change in hepatic fat (>1% change in MR PDFF), the optimal cutoff value of CAP change after treatment was selected as 38 dB/m with AUROC = 0.559 (Figs. 3A and B). Furthermore, when the CAP change was > 38 dB/m, the predictive value (positive predictive value) was 14/16 (87.5%), whereas when the CAP value was < 38 dB/m, the predictive value (negative predictive value) was 12/41 (29.3%). As a result, the accuracy of the method using CAP change was calculated as only 26/57 (46%). In addition, Cohen's kappa value, an index of the strength of agreement between 2 methods, was not significant (κ=0.11, *P* = .186) (Table 4).

**Table 4:**
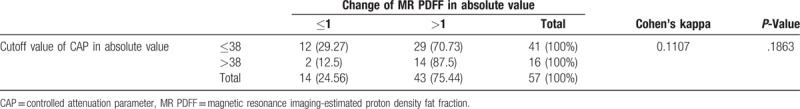
Predictive values of CAP change and agreement with MR PDFF change.

## 4 Discussion

In this study, we found that CAP is not sufficiently sensitive for monitoring hepatic steatosis change. When the CAP change after treatment was < 38 dB/m, the predictive value for hepatic steatosis change was only 12/41 (29.3%), implying that the predictive performance is very poor. Therefore, caution should be taken when interpreting the change in the CAP value. This is the 1st study on the use of CAP and MR PDFF to monitor longitudinal changes in hepatic steatosis.

The CAP has many advantages. First, in addition to being a noninvasive and painless method, it can also measure liver stiffness and steatosis at the same time. Second, CAP can be evaluated in samples of more liver parenchymal volume than liver biopsy samples. In this study, we found that CAP is a good diagnostic method for measuring hepatic steatosis. However, CAP has some limitations. First, MR PDFF is superior to CAP in diagnosing steatosis in patients with fatty liver. The CAP had a low AUROC of 0.85 (95% confidence interval [CI], 0.75–0.96) compared with MR PDFF, which detected any steatosis with an AUROC of 0.99 (95% CI, 0.98–1.00). The CAP is an accurate method for diagnosing any steatosis, but not at high dichotomized grades of steatosis compared with MR PDFF (AUROC 0.90 [95% CI, 0.82–0.97] vs. 0.70 [95% CI, 0.58–0.82]).^[11]^ There is also a lack of study on the role of CAP in the accurate measurement of hepatic steatosis change. It is important to assess hepatic steatosis and fibrosis in predicting prognosis in patients with fatty liver. It is also important to objectively measure hepatic steatosis before and after treatment, as various treatments for fatty liver are attempted. Recently, one study reported that hepatic steatosis estimated by using MR PDFF was correlated with histologic measures of steatosis and steatohepatitis.^[9]^ However, a simpler and more accurate method than MRI will be needed in the future, and the role of CAP in measuring hepatic fat change is expected to become important.

This study has some limitations. First, we measured hepatic fat change by using MR PDFF evaluation instead of liver biopsy. Although liver biopsy was not performed, recent studies showed that MR PDFF had nearly perfect correlation with biopsy data. ^[6,7,12]^ Moreover, although liver biopsy was used as a standard reference method, it also has many limitations such as sampling errors and intra- and inter-observer variations. Second, CAP measurement was performed only with the M probe of a FibroScan without an XL probe. Moreover, CAP had a relatively high measurement failure rate. In patients with a BMI of > 28 kg/m^2^ or higher, it is recommended to use an XL probe to reduce scan failures and to increase the reliability of hepatic steatosis measurement.^[13]^ Eight patients in this study were excluded owing to unreliable measurement results.^[13,14]^ Third, because of the small sample size of our study, large-scale investigations are need in the future.

In conclusion, CAP could have a diagnostic value for hepatic steatosis quantification and for assessing the changes in hepatic fat amount in clinical settings. However, careful interpretation of the steatosis change based on the CAP scores should be made when the absolute change value is < 38 dB/m in the clinical trial setting. More research is needed to determine the role of CAP in determining changes in hepatic steatosis.

## Author contributions

**Conceptualization:** Dae Won Jun, Bo-kyeong Kang.

**Data curation:** Sang Bong Ahn, Bo-kyeong Kang, Mimi Kim.

**Formal analysis:** Sang Bong Ahn, Bo-kyeong Kang, Mimi Kim, Misoo Chang, Eunwoo Nam.

**Funding acquisition:** Dae Won Jun.

**Investigation:** Sang Bong Ahn, Dae Won Jun.

**Methodology:** Sang Bong Ahn, Dae Won Jun, Bo-kyeong Kang, Mimi Kim.

**Resources:** Sang Bong Ahn, Dae Won Jun, Bo-kyeong Kang, Mimi Kim.

**Software:** Misoo Chang, Eunwoo Nam.

**Supervision:** Sang Bong Ahn, Dae Won Jun, Bo-kyeong Kang.

**Validation:** Misoo Chang, Eunwoo Nam.

**Writing – original draft:** Sang Bong Ahn.

**Writing – review and editing:** Dae Won Jun, Bo-kyeong Kang.

Sang Bong Ahn orcid: 0000-0001-7419-5259.

## References

[1] Kumar M Rastogi A Singh T Controlled attenuation parameter for non-invasive assessment of hepatic steatosis: does etiology affect performance? J Gastroenterol Hepatol 2013;28:1194–201. 23425053 10.1111/jgh.12134

[2] Chon YE Jung KS Kim SU Controlled attenuation parameter (CAP) for detection of hepatic steatosis in patients with chronic liver diseases: a prospective study of a native Korean population. Liver Int 2014;34:102–9. 24028214 10.1111/liv.12282

[3] Myers RP Pollett A Kirsch R Controlled attenuation parameter (CAP): a noninvasive method for the detection of hepatic steatosis based on transient elastography. Liver Int 2012;32:902–10. 22435761 10.1111/j.1478-3231.2012.02781.x

[4] Desai NK Harney S Raza R Comparison of controlled attenuation parameter and liver biopsy to assess hepatic steatosis in pediatric patients. J Pediatr 2016;173: 160-164 e161. 10.1016/j.jpeds.2016.03.021 PMC5105890 27039224

[5] Karlas T Petroff D Garnov N Non-invasive assessment of hepatic steatosis in patients with NAFLD using controlled attenuation parameter and 1H-MR spectroscopy. PLoS One 2014;9:e91987. 24637477 10.1371/journal.pone.0091987 PMC3956815

[6] Reeder SB Robson PM Yu H Quantification of hepatic steatosis with MRI: the effects of accurate fat spectral modeling. J Magn Reson Imaging 2009;29:1332–9. 19472390 10.1002/jmri.21751 PMC2689318

[7] Permutt Z Le TA Peterson MR Correlation between liver histology and novel magnetic resonance imaging in adult patients with non-alcoholic fatty liver disease - MRI accurately quantifies hepatic steatosis in NAFLD. Aliment Pharmacol Ther 2012;36:22–9. 22554256 10.1111/j.1365-2036.2012.05121.x PMC3437221

[8] Noureddin M Lam J Peterson MR Utility of magnetic resonance imaging versus histology for quantifying changes in liver fat in nonalcoholic fatty liver disease trials. Hepatology 2013;58:1930–40. 23696515 10.1002/hep.26455 PMC4819962

[9] Middleton MS Heba ER Hooker CA Agreement between magnetic resonance imaging proton density fat fraction measurements and pathologist-assigned steatosis grades of liver biopsies from adults with nonalcoholic steatohepatitis. Gastroenterology 2017;153:753–61. 28624576 10.1053/j.gastro.2017.06.005 PMC5695870

[10] Boursier J Zarski JP de Ledinghen V Determination of reliability criteria for liver stiffness evaluation by transient elastography. Hepatology 2013;57:1182–91. 22899556 10.1002/hep.25993

[11] Park CC Nguyen P Hernandez C Magnetic resonance elastography vs transient elastography in detection of fibrosis and noninvasive measurement of steatosis in patients with biopsy-proven nonalcoholic fatty liver disease. Gastroenterology 2017;152: 598-607 e592. 10.1053/j.gastro.2016.10.026 PMC5285304 27911262

[12] Imajo K Kessoku T Honda Y Magnetic resonance imaging more accurately classifies steatosis and fibrosis in patients with nonalcoholic fatty liver disease than transient elastography. Gastroenterology 2016;150: 626-637 e627. 10.1053/j.gastro.2015.11.048 26677985

[13] Sasso M Audiere S Kemgang A Liver steatosis assessed by controlled attenuation parameter (CAP) measured with the XL probe of the FibroScan: a pilot study assessing diagnostic accuracy. Ultrasound Med Biol 2016;42:92–103. 26386476 10.1016/j.ultrasmedbio.2015.08.008

[14] Wong VW Vergniol J Wong GL Liver stiffness measurement using XL probe in patients with nonalcoholic fatty liver disease. Am J Gastroenterol 2012;107:1862–71. 23032979 10.1038/ajg.2012.331

